# Flame-Retardant ABS Composites for 3D Printing: Synergistic Effects of Phosphorus-Based Additives

**DOI:** 10.3390/ma19142983

**Published:** 2026-07-10

**Authors:** Rafał Oliwa, Katarzyna Bulanda, Mariusz Oleksy

**Affiliations:** Department of Polymer Composites, Faculty of Chemistry, Rzeszow University of Technology, Al. Powstańców Warszawy 6, 35-029 Rzeszów, Poland; oliwa@prz.edu.pl (R.O.);

**Keywords:** ABS composites, fire behavior, flame retardancy, 3D printing

## Abstract

In this study, the effects of the type and content of phosphorus-based flame retardants, namely melamine polyphosphate (MPP) and aluminum diethylphosphinate (AlDPi), as well as their hybrid systems (MPP:AlDPi ratios of 1:1, 1:3, and 3:1), on the fire performance of acrylonitrile-butadiene-styrene (ABS) composites were investigated. The obtained results indicate that the synergistic action of MPP and AlDPi, including simultaneous inhibition of combustion in the gas phase and action in the condensed phase, leads to a significant improvement in the fire-retardant properties of ABS composites. For unmodified ABS, the peak Heat Release Rate (pHRR) and Total Heat Released (THR) values were 808.7 kW/m^2^ and 86.5 MJ/m^2^, respectively, while for the ABS/MPP_15/AlDPi_5, these values decreased to 292.9 kW/m^2^ and 32.3 MJ/m^2^. Simultaneously, the Effective Heat of Combustion (EHC) decreased from 22.3 to 15.5 MJ/kg, indicating inhibition of combustion processes in the gas phase. Fourier Transform Infrared Spectroscopy (FTIR) analysis of post-combustion residues (peaks 1280 and 1168 cm^−1^) confirmed the contribution of additives to the formation of phosphorous derivatives in the condensed phase. The hybrid system ABS/MPP_15/AlDPi_5 exhibited the most favorable fire performance in cone calorimeter tests, characterized by reduced heat release and fire growth parameters. This was confirmed by the calculated fire performance indicators, including Fire Growth Rate Index (FIGRA), Maximum Average Rate of Heat Emission (MARHE), Fire Potential Index (FPI), and Flame Retardancy Index (FRI).

## 1. Introduction

Acrylonitrile butadiene styrene (ABS) is one of the most commonly used thermoplastics in engineering applications, particularly in 3D printing by extrusion, due to its good mechanical properties and ease of processing [1,2,3,4]. However, its inherent flammability and tendency to drip significantly limit applications requiring stringent fire safety standards (e.g., high limiting oxygen index (LOI), UL-94 V-0, and low heat release) [3,4,5]. This challenge is particularly significant in additive manufacturing, where complex geometry, internal cavities, and high component integration can complicate fire risk assessment and heat and smoke dissipation in the event of ignition. In 3D printing with melted material, the layer-by-layer build-up process typically produces anisotropic structures with weak interlayer bonds, internal voids, and partially filled areas. This can lead to localized ignition, rapid flame propagation along interphase defects, and uneven heat and smoke release compared to parts formed by other methods such as injection molding. Furthermore, this may affect the relative importance of flame-retardant mechanisms occurring in the gas phase and the condensed phase [6,7]. The high flammability of ABS therefore requires material modification to improve fire safety [8].

One of the most effective methods for reducing polymer flammability is the physical modification with flame retardants. For regulatory, environmental, and technical reasons, the world has had to move away from brominated systems toward halogen-free systems, like phosphorus-based flame retardants in ABS [3,5,9,10]. Organophosphate families—phosphates, phosphonates, phosphinates, hypophosphites, and polyphosphates—have been studied alone or in multicomponent systems, often in combination with nitrogen-, silicon-, or mineral-based synergists, to balance fire resistance, smoke suppression, and mechanical properties [11,12,13,14,15,16,17,18]. In ABS, phosphorus species with higher oxidation states typically act primarily in the condensed phase, promoting char formation and swelling, while those with lower oxidation states are more active in the gas phase, releasing PO· radicals that quench combustion-promoting radicals.

For example, aluminum diethylphosphinate (AlDPi) is an organophosphonate with phosphorus in the +III oxidation state, which partially evaporates and releases diethylphosphinic acid, providing strong flame retardancy and fuel dilution in the gas phase, while a smaller fraction contributes to the formation of aluminum phosphate in the condensed phase. The lower valence of phosphorus in AlDPi promotes the formation of PO·/PO_2_· radicals during the decomposition of diethylphosphinic acid within a relatively narrow temperature range, which favors the dominant mechanism in the gas phase [19]. On the other hand, melamine polyphosphate (MPP) decomposes mainly in the condensed phase, releasing melamine and polyphosphate derivatives. The high oxidation state of phosphorus in the MPP polyphosphate backbone (+V) means that its degradation products are mainly acidic, highly oxidized phosphates, which catalyze dehydration and act as a binder, forming a more continuous, thermally stable barrier layer with a high phosphorus content [20]. Combining additives with complementary mechanisms has therefore been an important strategy for achieving high performance while maintaining moderate levels of additive content. According to the literature, when AlDPi is combined with polyphosphates and cyanurates, interactions according to Lewis acid–base theory may promote the formation of aluminum phosphate and related P–N-containing structures, leading to thicker, more homogeneous scale layers and simultaneous scavenging of gas-phase radicals, which significantly reduces PHRR, THR, and dripping in several matrices [21,22,23,24].

Strong evidence for synergy between different phosphorus functional groups in ABS has been published. Materials based on ammonium polyphosphate (APP) and aluminum diethylphosphinate (AlDPi) or related phosphinates can achieve UL-94 V-0 classification and LOI values above 28–30% with total flame retardant contents of approximately 20–25 wt%, along with a 70–75% reduction in peak heat release rate (pHRR) and significant reductions in fire growth rates [4,5,11,12,25]. Similar P–N and P–Si synergistic systems, including melamine salts, silicone rubbers, and phosphinate–silicone core–shell particles, have been shown to simultaneously improve fracture toughness, reduce pHRR and smoke generation, and enhance charcoal cohesion through combined vapor-phase inhibition and condensed-phase barrier formation [9,10,11,13,26]. More broadly, systematic evaluations of bromine-free flame retardant systems in ABS point to APP/AlDPi formulations, sometimes with expandable graphite or molybdenum-based smoke suppressants, as some of the most promising halogen-free solutions [27,28].

At the same time, several studies indicate that not all phosphate systems are equally effective in ABS or under conditions relevant to 3D printing. For example, in our previous work [6], the addition of tetrakis (2,6-dimethylphenyl)-m-phenylene biphosphate to extrusion 3D-printed ABS filaments increased the LOI only slightly and left the UL-94 rating at HB, acting almost exclusively in the gas phase and providing limited improvement in overall fire hazard metrics, despite some reduction in pHRR and MARHE. These findings highlight the importance of tailoring the oxidation state, chemical structure, and dispersion of phosphorus additives to the ABS matrix and processing, as well as quantitatively separating the gas and condensed phase contributions using cone calorimetry metrics (EHC, Heat Release Rate (HRR), total heat released (THR), FIGRA, FPI, and FRI) and residue analysis [3,4,8,9,29].

Recent work has focused on optimizing halogen-free, flame-retardant ABS through: finely tuned P–P or P–N synergies and hybrid swelling systems; incorporation of silicon-containing elastomers or POSS structures to improve char microstructure and mechanical strength; multifunctional nano- and hybrid additives (e.g., P/Ni-doped g-C_3_N_4_, zeolites) that combine catalytic charring, radical scavenging, and barrier effects; and bio-based or cork-derived phosphorus hybrids as more sustainable flame retardants [10,11,12,13,25,29]. However, the quantitative relationship between phosphorus oxidation state, action phase, and synergy indices in ABS composites specifically designed for 3D printing remains insufficiently elucidated.

In this context, this work investigates 3D extrusion-printed ABS composites containing melamine polyphosphate (MPP) and aluminum diethylphosphinate (AlDPi)—two phosphorus flame retardants with different oxidation states of phosphorus—used singly and in hybrid proportions ranging from 10 to 20 wt%. MPP is selected as a representative flame retardant with a high P/N ratio, which primarily promotes swelling and the formation of a cohesive char in the condensed phase, whereas AlDPi represents an organophosphonate with a lower degree of phosphorus oxidation, exhibiting pronounced flame retardancy in the gas phase, but a weaker charring effect. The contrast between the high phosphorus oxidation state in MPP and the reduced phosphorus oxidation state in AlDPi is therefore key to distinguishing the contributions of the condensed-phase and gas-phase mechanisms and to quantitatively understanding the synergy effects in 3D-printed ABS composites. Based on previous studies on the interaction of polyphosphates and phosphinates, the following hypotheses were proposed: MPP-based formulations will enhance protection in the condensed phase but provide limited flame retardancy in the gas phase; AlDPi-based formulations will act primarily in the gas phase and have limited charring capacity; and hybrid MPP/AlDPi will exhibit positive synergy indices, reflecting simultaneous action in both the gas and condensed phases, which is particularly beneficial for anisotropic, filled, 3D-printed ABS geometries.

By combining LOI and UL-94 testing with cone microcalorimetry, combustion residue analysis, and introducing a synergy effect index to emphasize gas and condensed phase contributions, this study aims to explain how phosphorus oxidation state and hybridization influence residue formation, heat release, and overall fire resistance of 3D-printed ABS parts.

## 2. Materials and Methods

### 2.1. Materials

Commercial granulate (Terluran HI-10 natural from Ineos Styrolution Group, Frankfurt am Main, Germany) designated as ABS was used as the polymer matrix. Melamine polyphosphate, MPP, and aluminum diethylphosphinate, AlDPi (WTH Walter Thieme Handel GmbH, Stade, Germany), were used as flame retardants.

The ABS compositions are summarized in Table 1.

### 2.2. Preparation of the Composites and Sample

The materials (ABS and flame retardants) were dried in a vacuum oven to remove moisture that could hinder the extrusion process (ABS—time: 4 h, temperature: 80 °C; flame retardants—time: 3 h, temperature: 100 °C).

**Figure 1:**
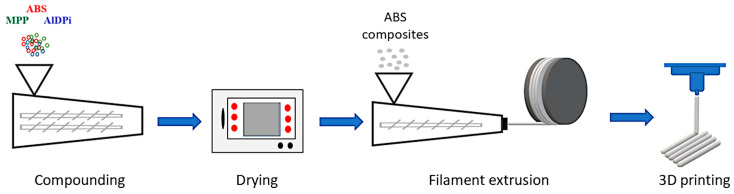
Flowchart for the preparation of ABS composites containing flame retardants.

According to the flowchart presented in Figure 1, the appropriate amounts of unmodified ABS granulate and the flame retardants MPP and AlDPi were mixed. The components were homogenized using a Coperion ZSK 18 ML twin-screw extruder (Coperion GmbH, Stuttgart, Germany) equipped with a pelletizing line. Process parameters: screw speed: 300 rpm, extruder output: 3 kg/h, and temperature range: 230–245 °C.

The resulting granulates were dried again in a vacuum oven at 80 °C for 3 h. From the resulting polymer composites, fibers with a diameter of approximately 1.75 ± 0.05 mm were obtained on a designed filament production line (Gamart SA, Jasło, Poland) at a temperature range of 225–240 °C. These values allowed for the production of a 1.75 mm diameter filament, suitable for 3D printing. The designed, proprietary production line is presented in our previous works [30,31].

It should be emphasized that both the unmodified ABS and all its composites obtained were prepared using the same manufacturing process, including all processing stages, i.e., extrusion and 3D printing. As a result, the unmodified ABS was used as a process control, which allowed for a reliable comparison of the results and a clear attribution of the observed changes in properties to the influence of the flame retardants used.

The appropriate samples in the form of a square of 100 mm × 100 mm × 4 mm, a beam of 125 mm × 12.7 mm × 4 mm, and a beam of 10 mm × 80 mm × 4 mm were designed using a CAD program. The file was saved with the “.stl” extension—a format readable by the ideaMaker (version 5.3.1 Beta)—3D printer control software. The composites were used to obtain samples needed for further testing on a 3D printer (Raise3D E2CF, Raise 3D Technologies, Shanghai, China) using FFF technology. The following 3D printing parameters were used: nozzle diameter 0.4 mm, layer height 0.2 mm, 100% infill with a ±45° pattern, extrusion temperature 275 °C, bed temperature 100 °C, and print speed 70 mm/s.

### 2.3. Method Characterization

The chemical structures of ABS and ABS composites after filament fabrication were characterized using an Alpha FTIR spectrometer (Bruker, Billerica, MA, USA) with an ATR attachment (attenuated total reflection). Three samples from each part were scanned 64 times each in the wavenumber range of 4000–400 cm^−1^. The analysis of the spectra obtained was carried out using the Opus software 6.5 (Bruker, Billerica, MA, USA).

The heat release rate (HRR in kW/m^2^) during combustion of the sample, as well as other parameters characterizing fire spread, were assessed using a mass loss cone calorimeter (FTT Ltd., East Grinstead, UK) in accordance with ISO 13927:2023 [32]. In the conducted research, a heat flux of 50 kW/m^2^ was selected, which is the most frequently used and allows for simulating conditions in the developing fire phase and ensures greater repeatability of results [33]. The distance from the ignition source was 25 mm. Printed samples measuring 100 mm × 100 mm × 4 mm were tested. The results are the arithmetic means of 3 tests for each type of composite.

The limiting oxygen index (LOI) for the 10 samples of each type of composite was determined according to EN ISO 4589 [34] at room temperature using equipment from Fire Testing Technology Ltd. (East Grinstead, UK). Printed samples measuring 10 mm × 80 mm × 4 mm were tested.

UL94 flammability testing was conducted in a test chamber manufactured by FTT Ltd. (East Grinstead, UK). Measurements were performed according to PN EN 60695-11-10 [35] using a vertically and horizontally positioned beam and a 25 mm high methane burner. Printed samples measuring 12.7 mm × 125 mm × 4 mm were tested. In the vertical test configuration, all test specimens burned completely, making it impossible to classify them according to the UL 94 criteria for that test setup. For this reason, further evaluation of the flame resistance of ABS composites was conducted in a horizontal configuration, which allowed for the determination of the burning rate and flammability class. Each type of test was performed on at least three samples from each series.

A thermal decomposition behavior test of the ABS composites was performed using a TGA/DSC thermogravimeter (METTLER Toledo, Albstadt, Germany), with the temperature ranging between 25 °C and 600 °C at a heating rate of 10 °C/min under a nitrogen atmosphere. For each type of composite, two measurements were made.

The chemical composition of the residue after mass loss cone calorimetry testing was determined using an Alpha FTIR Spectrometer (Bruker) for the infrared wavelength range of 4000–400 cm^−1^ using a powdered residue tablet in KBr.

## 3. Results and Discussion

### 3.1. Chemical Structure

**Figure 2:**
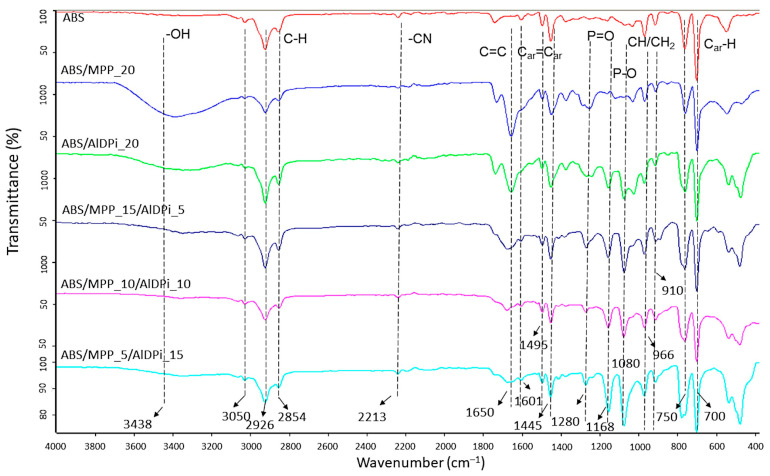
FT-IR spectra of a filament surface fabricated from ABS and ABS-based composites.

FTIR analysis was performed to identify the functional groups. Figure 2 shows that the FTIR spectrum of ABS contains characteristic absorption bands assigned to the individual components of the copolymer. The peaks at approximately 3050 cm^−1^, 2926 cm^−1^, and 2854 cm^−1^ correspond to stretching vibrations of C–H bonds, with the band above 3000 cm^−1^ associated with aromatic (unsaturated) C–H vibrations, while the bands at 2926 and 2854 cm^−1^ correspond to aliphatic C–H vibrations. The characteristic band at approximately 2213 cm^−1^ is attributed to stretching vibrations of the nitrile group (C≡N) derived from acrylonitrile units, which is one of the main identifying bands of ABS. The bands observed around 1601, 1445, and 1495 cm^−1^ correspond to stretching vibrations of the C=C bonds in the aromatic ring of styrene units. In turn, the signal at around 1650 cm^−1^ can be assigned to deformation of the C=C groups in poly(butadiene) [36]. In the lower wavenumber range, the band near 965 and 910 cm^−1^ corresponds to deformational vibrations of C–H and CH_2_, which are characteristic of butadiene units. In contrast, the peaks at around 750 cm^−1^ and 700 cm^−1^ are associated with out-of-plane vibrations of the aromatic C–H bonds of the benzene ring [37,38,39]. Additionally, new absorption bands are observed in the FTIR spectra of composites containing MPP and AlDPi flame retardants, indicating the presence of phosphorus compounds. A broad band around 3438 cm^−1^ is attributed to stretching vibrations of –OH groups, which are primarily associated with adsorbed water or hydroxyl groups present in the additive structure. The band at approximately 1280 cm^−1^ corresponds to stretching vibrations of P=O bonds, which are characteristic of phosphorus compounds. Meanwhile, the signals around 1080 cm^−1^ and 1168 cm^−1^ are attributed to diethylphosphinate anions PO_2_- [40,41].

### 3.2. Fire Behavior

**Figure 3:**
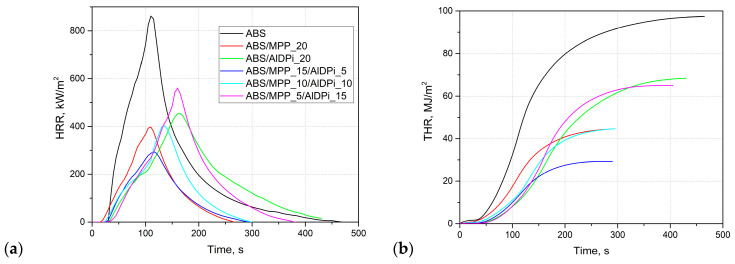
Representative curves: (**a**) HRR and (**b**) THR vs. time during flammability tests performed in the MLC calorimeter for ABS and ABS-based composites.

Combustion and potential fire hazard tests for ABS and ABS-based composites were conducted using a mass loss cone calorimeter. The results for individual combustion parameters were generated using the “MLC Calc” program, both numerically and graphically. Figure 3 illustrates the characteristic heat release rate (HRR) and total heat released (THR) curves for the tested materials, and Table 2 summarizes the main results obtained from their analysis.

The results clearly indicate that ABS modification with flame retardant additives significantly affects the flame resistance parameters of the materials. Unmodified ABS is characterized by the highest HRR (268.9 kW/m^2^), THR (86.5 MJ/m^2^), and pHRR (808.7 kW/m^2^) values, which indicate its high flammability and intense heat release during combustion [42,43]. The introduction of MPP leads to a significant reduction in combustion parameters, especially when 20% of the flame retardant is incorporated into the polymer matrix, HRR (90.4 kW/m^2^) and pHRR (408.1 kW/m^2^), which demonstrates the effectiveness of this flame retardant in reducing combustion intensity [12]. However, a shortening of the ignition time is observed (TTI up to 12 s), which may result from earlier decomposition of the additive and initiation of degradation processes, according to TGA data. In the case of AlDPi, the HRR (186.6 kW/m^2^) and pHRR (454.1 kW/m^2^) values are reduced to 20% additive content compared to ABS, but this reduction is less pronounced than for MPP. At the same time, these materials are characterized by higher PML values (99%), indicating a greater total mass loss during combustion. The most favorable flame retardancy properties were obtained for hybrid systems containing both additives. The ABS/MPP_15/AlDPi_5 composite exhibits the lowest pHRR value (292.9 kW/m^2^) and a significantly reduced THR value (32.3 MJ/m^2^), indicating a significant reduction in both the intensity and total amount of heat released.

The activity of the additives in the gas phase was analyzed based on changes in the effective heat of combustion (EHC), while their effect in the condensed phase was assessed by the post-combustion residue (PML). A decrease in the EHC value indicates inhibition of combustion processes in the flame, while a higher post-combustion residue indicates more intense formation of a char layer that limits heat and mass transfer.

The relationships presented in Table 2 were used to quantitatively evaluate the effect of phosphorus flame retardants on the properties of the resulting composites.

The following relationships were assumed:


(1) $$\mathrm{Gas phase activity}=1-\frac{{EHC}_{ABS+MPP}}{{EHC}_{ABS}}\;\mathrm{or}\;1-\frac{{EHC}_{ABS+AlDPi}}{{EHC}_{ABS}}\;\mathrm{or}\;1-\frac{{EHC}_{ABS+MPP+AlDPi}}{{EHC}_{ABS}}$$

(2) $$\mathrm{Charring effect/decomposition promotion}=1-\frac{{PML}_{ABS+MPP}}{{PML}_{ABS}}\;\mathrm{or}\;1-\frac{{PML}_{ABS+AlDPi}}{{PML}_{ABS}}\;\mathrm{or}\;1-\frac{{PML}_{ABS+MPP+AlDPi}}{{PML}_{ABS}}$$

(3) $$\mathrm{Protection}\;\mathrm{effect}=1-\frac{\frac{pHRR_{ABS+MPP}}{pHRR_{ABS}}}{\frac{THR_{ABS+MPP}}{THR_{ABS}}}\;\mathrm{or}\;1-\frac{\frac{pHRR_{ABS+AlDPi}}{pHRR_{ABS}}}{\frac{THR_{ABS+AlDPi}}{THR_{ABS}}}\;\mathrm{or}\;1-\frac{\frac{pHRR_{ABS+MPP+AlDPi}}{pHRR_{ABS}}}{\frac{THR_{ABS+MPP+AlDPi}}{THR_{ABS}}}$$

For ABS, the EHC value was 22.3 MJ/kg, and the post-combustion residue was relatively low (PML = 94.9%). The introduction of the MPP additive leads to a lower EHC parameter (reduction to 89.7% for ABS/MPP_20). At the same time, no significant increase in combustion residue was observed (PML = 93.5%), corresponding to a reduction in the release of flammable gases to only 98.5%. The THR (46.0 MJ/m^2^) was reduced to 53.56%, and pHRR (408.1 kW/m^2^) was reduced to 50.45% for ABS/MPP_20. According to calculations (89.7% × 98.5% × 57.1% = 50.5%), the effectiveness of MPP is due to the activity in the gas phase (10.3%) and the protective barrier effect (42.9%). However, the low final residue after combustion indicates its thermal instability and the decompositions during combustion. This is also evident in the THR vs. time graph, which shows a steadily increasing heat release rate until the material is completely burned out (Figure 2). In the case of composites with AlDPi, a similar relationship was observed. With the AlDPi loading, the EHC was reduced to 17.2–20.4 MJ/kg, along with very high PML values of 98–99%, which translates to minimal post-combustion residues [36]. As a result, this causes an increase in combustible gas emissions by up to 104%. The greatest reduction in the analyzed fire parameters was observed for the hybrid MPP/AlDPi systems. The hybrid systems, particularly ABS/MPP_15/AlDPi_5, showed the most favorable combination of reduced EHC, THR, and pHRR values, which was confirmed by the significant reduction in EHC (to 74.9%), THR (to 41.1%), and pHRR (to 36.0%). According to the calculated values (74.9% × 100.5% × 47.8% = 35.9%), both gas-phase activity and condensed-phase effects contribute to the reduction in THR and pHRR. However, as is the case with composites containing a single flame retardant, the residue formed is thermally unstable, leading to significant weight loss after combustion. In summary, the obtained results (Table 3) indicate that the common feature of the flame retardants used is the gas-phase activity of ABS composites, with AlDPi exhibiting higher activity in the gas phase, which is consistent with the literature [21]; however, this promotes the decomposition of the polymer matrix, resulting in a negative charring effect. The additional introduction of MPP in composites with a hybrid content of flame retardants improves the charring effect, which, despite its small proportion, in combination with the additional enhancement of activity in the gas phase, allows for the most valuable results to be obtained, particularly in the ABS/MPP_15/AlDPi_5 composite. This results from the interaction between flame retardants in accordance with Lewis acid–base theory, leading to more comprehensive material protection.

However, the THR index does not allow for a clear assessment of the composites’ fire resistance, and the test conditions in the cone microcalorimeter limit its ability to represent a real fire. The HRR as a function of time also does not reflect the actual flame spread across the material surface. Therefore, a set of additional indices [44,45,46,47] describing the course of the fire (Table 4) was used to assess the effectiveness of the applied MPP and AlDPi flame retardants:

- MARHE [kW/m^2^]—Maximum Average Rate of Heat Emission: The MARHE characterizes the potential spread of fire or flame. This value was read from ARHE (Average Rate of Heat Emission) graphs generated by the “MLC Calc” program.
- FIGRA [kW/m^2^·s]—Fire Growth Rate Index: The FIGRA determines the ability for fire to occur. It is defined as the maximum value of HRR over the time of occurrence of this value, as shown in the following formula:

(4) $$FIGRA=\frac{pHRR}{T\;pHRR}\;[kW/m^{2}\cdot s],$$

- FPI [m^2^·s/kW]—Fire Potential Index: The FPI is the assessment of the efficiency of the formation of a scale on the tested composites. It is defined as the time to ignition over the maximum HRR value:

(5) $$FPI=\frac{TTI}{pHRR}\;[m^{2}\cdot s/kW],$$

- FRI [−]—Flame Retardancy Index: The FRI is the quantitative assessment of the effectiveness of the flame retardants used. It is defined via the following relationship:

(6) $$FRI=\frac{{\left[THR\cdot \frac{pHRR}{TTI}\right]}_{\;ABS}}{{\left[THR\cdot \frac{pHRR}{TTI}\right]}_{ABS+flame\;retardant}}\;[-],$$

The obtained results of the calculations of the indicators are presented in Table 4.

**Figure 4:**
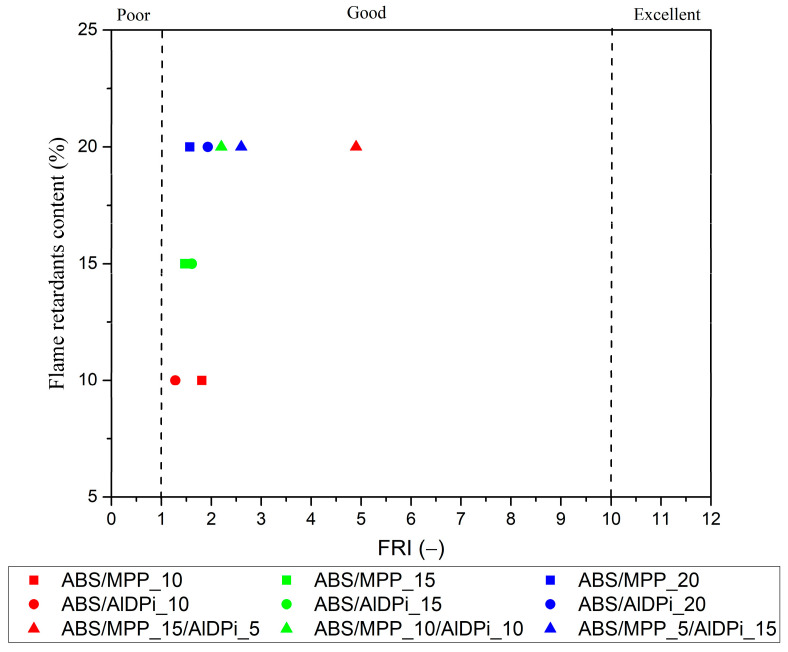
Relationship between the FRI index and the amount of flame retardants incorporated into ABS composites. Dashed lines indicate the classification ranges: poor, good, and excellent.

Analyzing the data (Table 4) regarding the MARHE index, it was found that the addition of flame retardants reduced the maximum average heat emission rate. This is consistent with the pHRR results as MARHE is directly related to HRR. Similar to the pHRR values among composites with a single flame retardant, lower MARHE values were obtained in the case of the MPP addition. The use of hybrid flame retardant content resulted in a reduction in MARHE compared to composites with MPP or AlDPi, except for the composite with AlDPi/MPP in a 2:1 ratio. As a result, the lowest MARHE value was found for the material with 15% MPP and 5% AlDPi. Similar correlations were obtained for the FIGRA parameter. However, the ABS/AlDPi composites showed lower values compared to the ABS/MPP composites. This is due to the combustion process of these materials, as recorded in the HRR plot as a function of time. Composites containing 20 wt% MPP reached their peak heat release rate in a shorter time (approx. 110 s), whereas for composites containing 20 wt% AlDPi, the fire development was slower, and the HRR peak was recorded after 160 s. However, once again, the lowest value was obtained for the ABS/MPP_15/AlDPi_5 composite (143.8 kW/m^2^), while the highest value was obtained for unmodified ABS (395.0 kW/m^2^). This indicates that this formulation is the most effective at reducing the intensity of the fire and delaying its spread. This also affects the pHRR/TTI values. Due to the lower stability of MPP observed in the TGA analysis, the ignition time of MPP-based composites is significantly shorter compared to AlDPi-based composites and unmodified ABS. The use of a hybrid content of flame retardants extends the ignition time, which results in lower pHRR/TTI values and a higher FPI value. The FPI index increases for most materials, reaching a maximum for ABS/MPP_15/AlDPi_5 (0.060 m^2^ s/kW), indicating improved ignition resistance. The FRI value increases with the addition of flame retardants, with the highest value obtained for the ABS/MPP_15/AlDPi_5 composite (4.89), indicating a more than four-fold improvement in fire-retardant properties compared to ABS. This is particularly visible in the graph of the relationship between the FRI and the amount of additives used (Figure 4). Based on the FRI classification (1–10), all modified materials are characterized by good effectiveness, with synergistic MPP/AlDPi systems demonstrating the highest effectiveness in reducing flammability.

**Figure 5:**
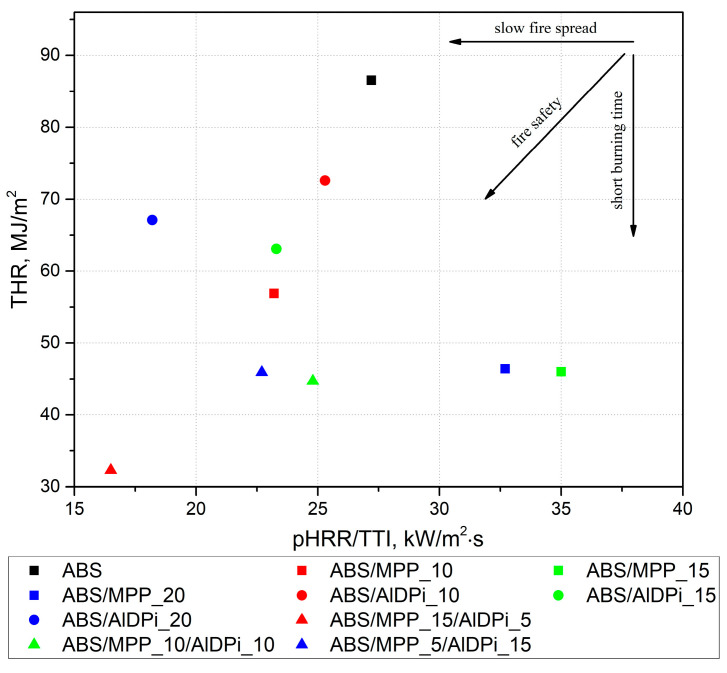
Petrella plots (THR vs. pHRR/TTI) for ABS-based composites containing different flame retardant systems (MPP, AlDPi). Arrows indicate directions of improved fire safety, including slower fire spread and shorter burning time.

To describe the dynamics of fire development, Petrella plots were prepared (Figure 5) [48]. ABS is characterized by the highest THR value (86.5 MJ/m^2^) and a relatively high pHRR/TTI (27.2 kW/m^2^·s), indicating an intense and rapidly developing fire. The introduction of MPP and AlDPi flame retardant additives causes a clear shift in the points toward lower values of both parameters, indicating improved fire protection properties. The most favorable results were obtained for MPP/AlDPi hybrid systems, particularly for the ABS/MPP_15/AlDPi_5 composite. The position of the points on the graph indicates that flame retardants reduce both the total heat release and the rate of fire development, which directly translates into greater fire safety and slower flame spread.

**Figure 6:**
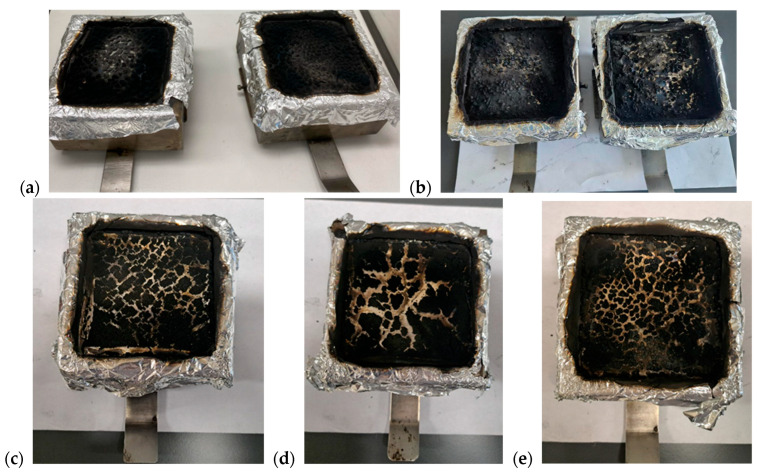
View of the samples after testing in a cone microcalorimeter: (**a**) ABS/MPP_20; (**b**) ABS/AlDPi_20; (**c**) ABS/MPP_15/AlDPi_5; (**d**) ABS/MPP_10/AlDPi_10; and (**e**) ABS/MPP_5/AlDPi_15.

Analysis of the char structure after testing in a cone microcalorimeter (Figure 6) confirms the previously discussed results, indicating a differentiated mechanism of action for the flame retardants used. The combustion residue indicates clear differences between the individual composites. Materials containing only the MPP flame retardant (Figure 6a) are characterized by a relatively uniform but porous char layer, which may indicate a limited ability to form a compact protective barrier. In the case of the second flame retardant, AlDPi (Figure 6b), the surface is more continuous, suggesting a more effective formation of a protective layer, limiting oxygen access and heat transfer. For mixed MPP/AlDPi systems (Figure 6c–e), a developed, cracked, and yet more ordered char structure is formed. This morphology is consistent with the improved fire-retardant performance observed for the hybrid systems. However, the exact contribution of gas-phase and condensed-phase mechanisms cannot be conclusively determined based solely on the present results.

### 3.3. Flammability

Collecting the LOI and UL94 test results in one place (Figure 6) allows us to clearly determine the effect of the type and content of flame retardant additives on the flammability of ABS and to demonstrate clear correlations between the two methods.

**Figure 7:**
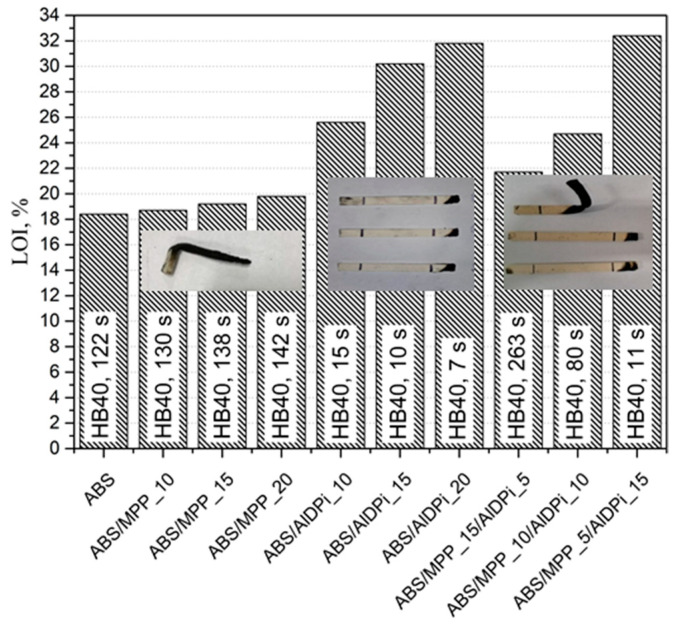
Limiting oxygen index (LOI) and UL-94 results for ABS and ABS containing flame retardant and samples viewed after the UL94 horizontal test.

ABS is characterized by a low oxygen index (18–19%) and a long combustion time (122 s), confirming its high flammability and lack of self-extinguishing properties (Figure 7). The introduction of MPP only slightly increases the LOI (20%), which also does not translate into an improvement of UL94 data. On the contrary, combustion times increase to approximately 130–142 s, indicating that this additive promotes stable, long-term combustion without effective flame extinction. A different trend can be observed for systems containing AlDPi. As its content increases, there is a simultaneous, significant increase in LOI (from approximately 25% for a 10% additive to over 30% for 15–20%) and a very significant reduction in combustion time in the UL94 test (from 15 s to as little as 7 s). In this case, a clear correlation is visible: an increase in the oxygen index directly translates into improved self-extinguishing ability [48].

In hybrid MPP/AlDPi systems, these relationships are more complex and strongly dependent on the component ratios. At a high content ratio of MPP compared to AlDPi (3:1), a relatively low LOI (22%) and a very long combustion time (263 s) are observed, indicating the unfavorable effects of this component in such proportions. When the AlDPi:MPP content ratio is 1:1 and 3:1, both the LOI and the UL94 results were improved—the oxygen index increases (24–33%) and the combustion time is significantly shortened to 80 and 11 s, respectively. The best properties are achieved by the system with a dominant AlDPi content, where a high LOI corresponds to rapid flame extinction.

To quantify whether two flame retardants perform better together than separately, the synergy effect index (SE) was calculated (Table 5) [49].

The following formula was used to determine SE:

(7) $$SE{\left(X\right)}_{x+y}=const=\;\frac{\;X_{ABS}-X_{ABS+(MPP+AlDPi)}}{\frac{x_{MPP}}{x_{MPP}+y_{AlDPi}}\left(X_{ABS}-X_{ABS+MPP}\right)+\frac{y_{AlDPi}}{x_{MPP}+y_{AlDPi}}(X_{ABS}-X_{ABS+AlDPi})}$$

where X—parameter value (pHRR, FRI, and LOI, respectively); x, y—flame retardant content, %.

On the basis of the obtained results of the synergy effect index, it was found that the interaction between MPP and AlDPi depends on the adopted evaluation parameter as well as their proportions in the composite. In terms of the ability to suppress fire spread and slow its growth, as represented by the pHRR parameter, a synergistic effect was observed between MPP and AlDPi at content ratios of 3:1 and 1:1. This effect results from the comprehensive action of the flame retardants in this combination, i.e., their effect in the gas phase, which is supported by charring in the condensed phase—a factor that is highly significant in slowing down fire kinetics. The use of a hybrid content of flame retardants, regardless of the ratio, also resulted in a synergistic effect in terms of reducing the fire load, as evidenced by the SE values for the index describing the effectiveness of the flame retardants (FRI). The highest effectiveness of MPP and AlDPi flame retardants was again confirmed at a 3:1 content ratio (SE = 6.24). Completely different SE results were obtained for the LOI index, which characterizes the composites’ resistance to a small flame. Only the ABS/MPP_15/AlDPi_5 composite exhibited a synergistic effect (SE = 1.35). This is due to the lower heat flux acting on the sample during the measurement, which favors the effectiveness of AlDPi in the gas phase and its evaporation [42]. The opposite effect applies to MPP, whose effectiveness increases at higher radiation intensities and is related to its action in the condensed phase.

### 3.4. Analysis of Flame Retardant Mechanism

#### 3.4.1. Thermogravimetric Analysis

**Figure 8:**
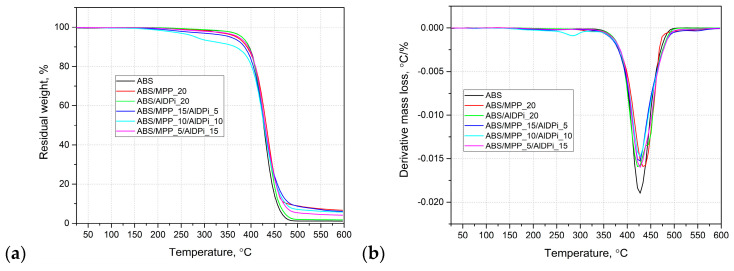
Mass change curves (**a**) and mass change derivative curves (**b**) of ABS and ABS-based composites.

Thermogravimetric analysis conducted in a nitrogen atmosphere determined the effect of MPP, AlDPi, and their hybrid content on the thermal stability of ABS composites. The obtained TG curves (Figure 8a) allowed for determining the 5% mass loss temperature (T5%), while the DTG curves (Figure 8b) allowed for determining the maximum degradation rate temperatures (Tmax) and the corresponding mass changes (Δm). Additionally, the mass residue at 600 °C (M600) and the thermal resistance index (THRI) were determined. All results are summarized in Table 6, which also includes the results for the flame retardants alone (MPP and AlDPi).

The analysis of the results indicates that ABS undergoes single-stage degradation (Tmax = 428.2 °C), which is consistent with the literature data [50,51]. The addition of MPP and MPP/AlDPi systems introduces a multi-stage degradation pattern, while the composite with only AlDPi maintains a process similar to single-stage degradation. Interestingly, analysis of the additives alone indicates significantly different degradation mechanisms. MPP is characterized by multi-stage degradation (five stages in the range of 192–542 °C). In contrast, AlDPi undergoes single-stage degradation at high temperature (Tmax = 462.1 °C) and leaves a significant amount of residue (10.2%), confirming its high thermal stability. The T5% temperature varies depending on the composite composition. Compared to ABS (386.4 °C), most modified materials show a reduction in this parameter, particularly for the ABS/MPP_15/AlDPi_5 system (279.7 °C), indicating an earlier initiation of degradation processes. The low T5% value for MPP (177.9 °C) suggests its early thermal activation, which may favor gas-phase operation. In the case of multi-stage composites, the first stage of degradation occurs at lower temperatures (230–320 °C) and is associated with the decomposition of phosphorus additives, while the next stage (423–437 °C) corresponds to the degradation of the ABS matrix.

Significant differences are also observed in the amount of residues after degradation. Unmodified ABS is characterized by no residue (0.0%), while the introduction of additives leads to an increase in M600, particularly for ABS/MPP_20 (6.7%) and ABS/MPP_5/AlDPi_15 (6.1%). The increased amount of residues confirms the contribution of the additives to the formation of the char layer, which is consistent with their behavior as fillers alone.

The behavior of composites in the initial stage of degradation is also reflected by the heat resistance index temperature (THRI). The THRI was calculated based on the equation in [41], using the temperatures at which 5% and 30% mass loss occurs:

(8) $$T_{HRI}=0.69\cdot \left[T_{5\%}+0.6\cdot \left(T_{30\%}-T_{5\%}\right)\right]$$

The THRI indicates the stability of the primary residue formed in the initial stage of degradation.

The THRI values range from 248.0 °C to 278.8 °C. The highest value was recorded for the ABS/MPP_20 composite, while the lowest was for the ABS/MPP_10/AlDPi_10 system, confirming its reduced thermal stability in the initial stage of degradation. At the same time, the THRI values for most composites remain similar to those for ABS, which confirms the low thermal stability of the formed residue.

#### 3.4.2. Char Residue Analysis

**Figure 9:**
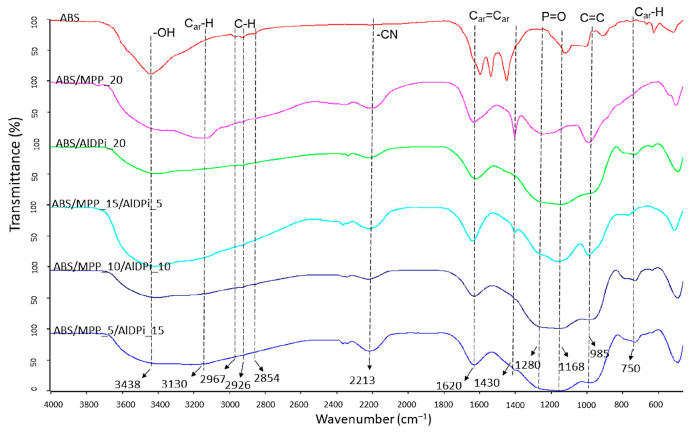
FT-IR spectra of char residue after the mass loss calorimeter test of ABS and ABS composites.

The FTIR spectra of post-combustion residues, shown in Figure 9, indicate significant structural changes in the tested materials. Compared to the samples before combustion (Figure 2), a significant weakening of the bands characteristic of aliphatic C–H groups in the 2926 and 2854 cm^−1^ ranges is observed, indicating degradation of the polymer chains. Changes can also be observed at approximately 3438 cm^−1^, where a broad band attributed to –OH groups becomes visible, which becomes flattened, which may be related to the presence of oxidation products and charred structures. The transmittance results of the post-combustion materials also contain peaks characteristic of aromatic and charred structures, particularly in the region around 1620 cm^−1^ (C=C vibrations in aromatic systems), indicating the presence of a stable char layer. Additionally, distinct peaks at 1430 cm^−1^, as well as 985 and 750 cm^−1^, associated with aromatic ring deformations, are observed. In samples containing flame retardants with phosphorus additives, the peaks in the range of approximately 1280 cm^−1^ and 1168 cm^−1^ are particularly significant, which can be attributed to P=O and P–O–C/P–O–P bond vibrations, respectively, confirming the participation of phosphorus compounds in the formation of the layer in the condensed phase [52]. The most pronounced peaks in this range are observed for systems containing both MPP and AlDPi, suggesting a greater contribution of phosphorus-containing structures in the combustion residue. The results are consistent with the improved fire performance observed for the hybrid systems.

## 4. Conclusions

The aim of this study was to develop ABS composites with improved fire resistance through the use of phosphorus-based flame retardants: melamine polyphosphate (MPP), aluminum diethylphosphinate (AlDPi), and their hybrid systems. The results confirmed that both individual additives and their hybrid systems lead to an improvement in the flammability properties of ABS composites. It was also demonstrated that the effectiveness of the hybrid systems is strongly dependent on the relative proportions of MPP and AlDPi.

The results suggest that the action of the flame retardants used includes both processes that take place in the gas phase and in the condensed phase. Thermal analysis and the characterization of combustion residues indicate the role of phosphorus-containing structures in the formation of protective residues during combustion. At the same time, the available results do not allow for a clear determination of the dominant mechanism responsible for the observed improvement in fire resistance.

Among the materials tested, the most favorable properties were obtained for hybrid systems, particularly in terms of limiting heat release and fire spread. At the same time, it was demonstrated that the assessment of the effectiveness of flame-retardant agents and the synergistic effect depends on the fire parameter used. Synergistic effects were clearly visible for selected parameters determined by the cone calorimeter method, which measures flame spread rate, whereas they did not occur to the same extent in the case of resistance to small flames (oxygen index).

The thermal behavior of hybrid systems results from the overlap of degradation processes in the flame retardants and the ABS matrix, indicating the complex nature of the interactions occurring during combustion. The results confirm that appropriately selected MPP/AlDPi hybrid systems represent a promising strategy for improving the fire properties of ABS composites intended for use in additive manufacturing technologies.

## Figures and Tables

**Table 1:** ABS compositions with flame retardants.

Sample Symbol	ABS Content (wt.%)	MPP Content (wt.%)	AlDPi Content (wt.%)
ABS	100	-	-
ABS/MPP_10	90	10	-
ABS/MPP_15	85	15	-
ABS/MPP_20	80	20	-
ABS/AlDPi_10	90	-	10
ABS/AlDPi_15	85	-	15
ABS/AlDPi_20	80	-	20
ABS/MPP_15/AlDPi_5	80	15	5
ABS/MPP_10/AlDPi_10	80	10	10
ABS/MPP_5/AlDPi_15	80	5	15

**Table 2:** Summary of average results of the main indices obtained from the cone microcalorimeter test.

Sample Symbol	TTI [s]	HRR [kW/m^2^]	pHRR [kW/m^2^]	THR [MJ/m^2^]	EHC [MJ/kg]	PML [%]
ABS	30.0 ± 2.8	268.9 ± 4.5	808.7 ± 83.8	86.5 ± 10.8	22.3 ± 3.0	94.9 ± 2.2
ABS/MPP_10	20.0 ± 0.5	119.8 ± 8.3	475.8 ± 27.5	56.9 ± 3.3	19.6 ± 0.2	96.8 ± 1.1
ABS/MPP_15	12.0 ± 0.5	189.3 ± 5.7	436.9 ± 3.8	46.0 ± 2.8	20.6 ± 1.4	96.1 ± 0.2
ABS/MPP_20	12.0 ± 0.5	90.4 ± 15.3	408.1 ± 10.9	46.4 ± 2.2	20.0 ± 2.7	93.5 ± 1.7
ABS/AlDPi_10	21.0 ± 1.0	220.4 ± 10.8	531.5 ± 5.8	72.6 ± 3.7	20.4 ± 3.1	99.0 ± 0.4
ABS/AlDPi_15	25.0 ± 1.0	187.9 ± 16.2	583.4 ± 38.1	63.1 ± 1.2	17.2 ± 0.5	98.9 ± 0.1
ABS/AlDPi_20	25.0 ± 0.5	186.6 ± 9.3	454.1 ± 15.2	67.1 ± 4.2	17.7 ± 1.5	98.8 ± 1.7
ABS/MPP_15/AlDPi_5	19.0 ± 4.5	118.1 ± 15.7	292.9 ± 1.8	32.3 ± 3.3	15.5 ± 1.3	94.1 ± 1.4
ABS/MPP_10/AlDPi_10	16.0 ± 2.5	133.9 ± 7.1	396.5 ± 7.3	44.7 ± 0.5	18.1 ± 0.8	93.3 ± 0.3
ABS/MPP_5/AlDPi_15	23.0 ± 3.0	146.5 ± 19.3	506.9 ± 43.7	46.0 ± 10.7	23.8 ± 7.3	94.9 ± 1.6

± standard deviation. HRR—Heat Release Rate; pHRR—peak Heat Release Rate; T pHRR—Time of peak Heat Release Rate; THR—Total Heat Released; TTI—Time to Ignition; EHC—Effective Heat of Combustion; and PML—Percentage Mass Loss.

**Table 3:** The effect of flame retardants.

Sample Symbol	Effect of Flame Retardants, %		
	Inhibition of Combustion in the Gas Phase	Charring (+)/Decomposition (−) in the Condensed Phase	Formation of a Protective Layer
ABS/MPP_10	12.0	−1.9	10.5
ABS/MPP_15	7.6	−1.2	−1.6
ABS/MPP_20	10.3	1.5	5.8
ABS/AlDPi_10	8.5	−4.3	21.7
ABS/AlDPi_15	22.9	−4.2	1.1
ABS/AlDPi_20	20.6	−4.1	27.6
ABS/MPP_15/AlDPi_5	30.7	0.9	2.9
ABS/MPP_10/AlDPi_10	18.8	1.7	5.1
ABS/MPP_5/AlDPi_15	−6.7	0.1	−18.1

**Table 4:** Summary of average results of calculated indicators.

Sample Symbol	MARHE [kW/m^2^]	FIGRA [kW/m^2^·s]	pHRR/TTI [kW/m^2^·s]	FPI [m^2^·s/kW]	FRI [−]
ABS	395.0 ± 62.2	7.07 ± 1.16	27.2 ± 5.4	0.037 ± 0.010	1.00 ± 0.33
ABS/MPP_10	227.7 ± 12.2	3.80 ± 0.07	23.4 ± 1.9	0.043 ± 0.004	1.81 ± 0.25
ABS/MPP_15	197.6 ± 2.7	3.89 ± 0.05	35.0 ± 1.1	0.029 ± 0.001	1.47 ± 0.04
ABS/MPP_20	195.7 ± 4.6	3.90 ± 0.29	32.7 ± 2.2	0.031 ± 0.002	1.57 ± 0.18
ABS/AlDPi_10	250.3 ± 3.1	3.43 ± 0.16	25.3 ± 1.3	0.040 ± 0.004	1.28 ± 0.16
ABS/AlDPi_15	226.5 ± 5.9	3.64 ± 0.19	23.3 ± 1.0	0.043 ± 0.055	1.61 ± 0.10
ABS/AlDPi_20	211.8 ± 7.3	2.75 ± 0.17	18.2 ± 2.0	0.055 ± 0.006	1.92 ± 0.41
ABS/MPP_15/AlDPi_5	143.8 ± 5.7	2.49 ± 0.07	16.5 ± 4.3	0.060 ± 0.017	4.89 ± 1.72
ABS/MPP_10/AlDPi_10	180.0 ± 2.8	2.99 ± 0.00	24.8 ± 0.5	0.040 ± 0.001	2.13 ± 0.02
ABS/MPP_5/AlDPi_15	209.6 ± 20.4	5.24 ± 0.73	22.7 ± 4.0	0.050 ± 0.008	2.59 ± 0.64

± standard deviation. MARHE—Maximum Average Rate of Heat Emission; FIGRA—Fire Growth Rate Index; pHRR/TTI—peak Heat Release Rate/Total Heat Released; FPI—Fire Potential Index; and FRI—Flame Retardancy Index.

**Table 5:** Synergy effect index.

Sample Symbol	Synergy Effect Index for Selected Flammability Indicators		
	SE (pHRR)	SE (FRI)	SE (LOI)
ABS/MPP_15/AlDPi_5	1.33	6.24	0.75
ABS/MPP_10/AlDPi_10	1.09	1.53	0.81
ABS/MPP_5/AlDPi_15	0.82	1.94	1.35

**Table 6:** TGA data of ABS and ABS composites.

Sample Symbol	T_5%_ [°C]	T_max 1_/T_max 2_/T_max 3_/T_max 4_/T_max 5_ [°C]	Δm_1_/Δm_2_/Δm_3_/Δm_4_/Δm_5_ [%]	M_600_ [%]	T_HRI_ [°C]
MPP	177.9	192.0/272.1/399.8/483.5/541.8	16.8/12.0/17.2/14.4/11.6	13.3	-
AlDPi	418.2	462.1	78.4	10.2	-
ABS	386.4	428.2	98.5	0.0	278.8
ABS/MPP_20	370.1	319.2/437.5	2.68/90.4	6.7	275.6
ABS/AlDPi_20	379.9	423.3	97.9	1.8	277.0
ABS/MPP_15%/AlDPi_5	279.7	283.1/426.3	8.0/86.1	5.8	248.0
ABS/MPP_10%/AlDPi_10	372.4	426.2	95.6	4.2	276.2
ABS/MPP_5%/AlDPi_15	356.6	230.5/426.6/548.4	2.96/88.9/1.72	6.1	269.6

## Data Availability

The data presented in this study are available upon request from the corresponding author.
